# Prevalence of Human Papilloma Virus Sub Genotypes following Head and Neck Squamous Cell Carcinomas in Asian Continent, A Systematic Review Article 

**DOI:** 10.31557/APJCP.2019.20.11.3269

**Published:** 2019

**Authors:** Naeem Bukhari, Joe P Joseph, Joseph Hussain, Muhammad Adeeb Muhammad Adeeb, Marcel Jose Yibirin Wakim, Esam Bashir Yahya, Amina Arif, Afshan Saleem, Nadeem Sharif

**Affiliations:** 1 *Centre for Human Genetics, Hazara University Mansehra, *; 4 *Department of Zoology, Women University of Azad Jammu and Kashmir, Bagh, *; 7 *Faculty of Life Sciences, University of Central Punjab,*; 8 *Department of Microbiology, *; 9 *Department of Medical Laboratory Technology, University of Haripur, Pakistan, *; 2 *Department of Physiology and Biophysics, Case Western Reserve University School of Medicine, USA,*; 3 *Department of Food Science and Engineering, University of Technology PR China, *; 5 *Internal Medicine Department, Central University of Venezuela, Venezuela, *; 6 *Department of Microbiology, Faculty of Science, Al Asmarya Islamic University, Zliten, Libya.*

**Keywords:** HPV, HNSCC, South Asia, Central Asia, Middle East

## Abstract

**Objectives:**

In current era of blue brain intelligence and technology access at ease, standardization of disease etiology demands extensive research to drop-down human papilloma virus associated head and neck squamous cell carcinomas impact at large. Present retrospection aims to estimate comparative association of human papilloma virus sub-genotypes in head and neck squamous cell carcinomas, critical analysis of existing research gap, treatment progress, co-infection, gender association, national status and challenges following Human papilloma virus led head and neck squamous cell carcinomas among world largest continent.

**Background:**

Head and neck squamous cell carcinomas are not just like malignancies of uterine cervix, lymph nodes and breast cancers. Human papilloma virus led head and neck squamous cell carcinomas treatment directly impact Central nervous system in humans. Intriguingly, human papilloma virus mediated immune response increases patient survival, which indirectly transmit human papilloma virus in future generations and act as a potential threat developing neurogenic disorders.

**Methods:**

An objective based search strategy, following comprehensive and specific search approaches were made to retrieve recent 12 years research data from five different NCBI databases. Out of 300 shortlisted articles, only 24 principal studies met the inclusion criteria.

**Results:**

Highest human papilloma virus prevalence (10.42 %) was found in South Asia, 5.8 % in South East Asia, 5.7 % East Asia, 2.5% in west Asia and no relevant updated data was found from central Asian continent. Highest prevalence (10%) of HPV genotype-16 was recorded in Asia among 3, 710 enrolled cases including 2201 males, 1149 females and 360 cases of unknown gender. While undifferentiated multiple HPV genotype prevalence was 5.5 % (204 cases). Lowest percentage of HPV sub-types 68, 72, 57, 39 were recorded respectively. Pakistan ranked top reporting highest number of HPV-16 cases, Taiwan HPV-18, India HPV-31, Japan HPV-35 and Singapore in HPV-16 and HPV-18 co-infection rates respectively.

**Conclusions:**

Exact prevalence of HPV associated head and neck squamous cell carcinomas among Asian population is still debatable. Due to higher heterogeneity (P< 0.00001), I^2^ = 81-88% at 95 % confidence interval), non-availability and limitations of reported studies from Asian sub-continents especially central Asia, western Asia and from south and south east Asia demand large scale collaborative research culture to standardize head and neck squamous cell carcinomas aetiology.

## Introduction

Classically HPV led squamous cell carcinomas of head and neck arise from oropharynx impacting multiple sites including, lip, tongue, eye, chin and nasopharynx as well (Syrjanen et al., 1983). Globally HNSCCs are the sixth most common deadly malignancies, causing 350,000 deaths each year with annual incidence of 600,000 reported cases (Ferlay et al., 2010). While, study of National statistics office in United Kingdom reported 7,745 cases each year (Office for National Statistics, 2009). Specifically, Oropharyngeal and tongue malignancies are predominant cancers in west. However, in general HNSCC a heterogenous group of tumours are shortly reported from South Asia instead of entire Asian continent (Bhurgri et al., 2006). According to Faheem A et al study in 2009, HNSCC is considered second most prevalent type of cancer in Pakistani subjects alone with recorded incidence of 40.1% (Bhurgri et al., 2006; Faheem et al., 2009). Commencing comparative clinical aetiology of HNSCCs from Europe based case control studies. About (70-75%) HNSCCs cases are synergistically associated with tobacco and alcohol consumption accompanied by familial history (Hashibe et al., 2009; McKay et al., 2011). Whereas, (10-15%) HNSSCs cases are associated with decreased intake of fruit and vegetables (Chuang et al., 2012; De Feo et al., 2008). In contrast to European countries, betel-quid chewing also plays a major role in the development of malignant tumours focusing Asian subjects (Chen et al., 2008). Molecular biology of Head and neck carcinomas revealed that genetic mutations and chromosomal abnormalities, especially mutation of p53 gene involvement in development of head and neck cancers (Leemans et al., 2011). Recent progression in research trends indicated decline in alcohol and tobacco induced HNSCCs. Whereases, Prevalence of HPV induced HNSCCs is increasing. Another supporting evidence suggest higher chances of HPV transmission during sexual behaviour in comparison to tobacco and alcohol consumption (Chaturvedi et al., 2011; Gillison., et al 2012). Instead of HPV-genotypes independently, co-infection with Epstein Barr virus also errand development of nasopharyngeal carcinomas (Termine et al., 2008). Classical research reported 95% prevalence of HPV in cervical carcinomas and 12.8%-59.9 % association of HPV-Subtypes in HNSCCs, including HPV-35, HPV-33, HPV-31, HPV-18 and HPV-16. Which clearly highlights existing research gap focusing HPV subtype specific prevalence and exact role following HNSCCs (Syrjanen et al., 1983; zur et al., 2009; Westra et al., 2009; Liebertz et al., 2010; Nasman et al., 2009; Ha et al., 2009). HPV led HNSCCs require progressive and meticulous treatment measures. After critical care and monitoring only five-year survival rate of 68% was possibly achieved yet. While delayed tumour detection and improper treatment may impact nervous system in humans. Intriguingly, HPV mediated immune response increases patient survival, which indirectly transmit HPV in future generations and act as a potential threat developing neurogenic disorders (Pai et al., 2009; Howlader et al., 2009). 

Present retrospection aims to estimate comparative association of HPV sub-genotypes in head and neck squamous cell carcinomas among world largest continent. The main objectives include to critically analyse existing research gap, epigenetic treatment progress, multiple genotype co-infection, gender association, national status and challenges following HPV related head and neck squamous cell carcinomas among Asian people. 

## Materials and Methods

*Database search strategy*

An objective based search strategy, following comprehensive and specific search approaches to retrieve recent 12.5-year research data from five different NCBI databases including PubMed, MeSH, PubMed Central, NLM Catalog and Bookshelf. 

*Inclusion and exclusion Criteria*

The articles published in English language focusing human subject only were considered. Criteria followed include recent 12.5-year studies reported from five different geographic regions of Asia. Studies who had significantly explained the role of any sub-genotype of HPV in head and neck cancer. While, studies contradicting above mentioned inclusion criteria were considered ineligible and excluded. Detailed inclusion and exclusion criteria are mentioned in Table 1.

*Studies Selection*

By Specific approach overall, Principal author and third co-author shortlisted n = 300 abstracts from NCBI databases, including n = 236 duplicate and older than Jan-2009 were excluded for further analysis. From above mentioned 300 citations, only 64 full text articles were found. But, Due to irrelevance with inclusion criteria 40 full text articles were excluded. However, 24 principal studies were focussed and critically analyzed Table 2. 

*Data Retrieval and Risk Measures*

Two co-investigators belonging from another country focused on data review and validation in collaboration with principal author for each included study. Country of study, first author, year of publication, type/site of lesion, number of cases included. Specific HPV type and overall prevalence of HPV in head and neck cancer infection was also studied. Prevalence estimation was done as total number of patient positive for specific HPV-subtype divided by total number of HNSCC patients. The accuracy of data search was counter checked by a second researcher, Further, Omissions; errors were resolved by sharing it with another co-investigator from another territory. 

*Statistical Analysis*

Statistical analysis was done by using Revman 5.30, Prisma and Microsoft Excel 2010 multiple tools. 

*Characteristics of Meta-Analysis*

Meta-analysis concentrating number of included studies from a country, patient selection, mean age, method followed, and relative outcomes as described in (Table 3) from 24 eligible studies. Khovidhunkit et al., (2008) reported only one patient with non-specific HPV out of 65 oral squamous cell carcinomas cases. Luo et al., (2007) examined 51 oral squamous cell carcinoma biopsies and reported 25 % high risk HPV positive cases. HPV-16 and HPV-18 were more prevalent than HPV-33 and HPV-52. Similar study carried by Li-Ang Lee et al., in (2012) reported increasing trend of high-risk HPV in Taiwan. Akhtar et al., (2013) examined 34 oral squamous cell carcinoma confirmed patients. author followed PCR method for detection of HPV subtypes highlighting no one person was affected by any specific high-risk HPV type among Bengali patients. Zhang et al., (2006) highlighted HPV association in oral squamous cell carcinomas including high risk HPV-16 and HPV-18 were positive among 18 patients out of total 63 confirmed cases. In 2012 two similar studies were carried out, one on healthy patient saliva and other on Oral squamous cell carcinomas patients reported 6.1 % and 3.9 % high HPV-6, HPV-18, HPV-66 and HPV-16, HPV-18, HPV-33, HPV-57 respectively (Hafed et al., 2012; Seifi et al., 2013). Another past study carried by Kermani I et al in 2012 reported 42.8% patients with high risk HPV-16 and HPV-18. He examined fourteen different Oro-pharyngeal, hypo-pharyngeal and laryngeal carcinoma patients. Maruyama H et al., 2014 reported 34.4 % specific type HPV 16, 18, 33, 35, 58 respectively among 163 confirmed patients of oropharynx. A recent study reported 20.4% high risk HPV-16 among 206 oral squamous cell carcinomas patients (Kerishnan et al., 2016). Baig et al., (2012) reported 47 HPV patients with high risk HPV-16 and HPV-18. They examined oral submucosal tissues from 262 Gutkka addict persons and HPV was present in 20 % patients. Na-Kyung Ryoo et al., 2013 tested 54 patients of retinoblastoma, found all patients were HPV negative. Another same country study conducted by Lee et al., (2010) on oral tongue lesions reported 36% non-specific HPV positive cases. Their Data showed 13 patients were HPV positive. Gunasekera et al., (2015) reported 46% high risk HPV-16 and HPV-18. Total 78 oropharyngeal carcinoma biopsies were included in study. In total 65 laryngeal and hypo-laryngeal carcinoma for high risk HPV-16. Only 27 (41.5%) samples were positive for HPV-16. Two studies conducted by Hafed et al., (2012) and Mansour et al., (2012) reported that high risk HPV-16 and HPV-18 were 23.52% and 86% respectively in tumour samples. Due to limited number of studies and underdeveloped infrastructure only one recent study conducted by Jalouli et al., (2012) reported that 20% HPV were positive in oral squamous cell carcinomas. 

## Results

Based on purely two objective based search strategies including comprehensive and specific quality data retrieval approaches. overall 56,302 search results, 10,999 were reported from south Asia, 2,346 from middle East, 1,710 from central Asia, 1,229 from East Asia and only 17 search results from western Asia were found from recent eleven year published sources (Table 4). Our specific approach was based upon shortlisting relevant studies reported from each of five Asian regions. 

**Figure 1:**
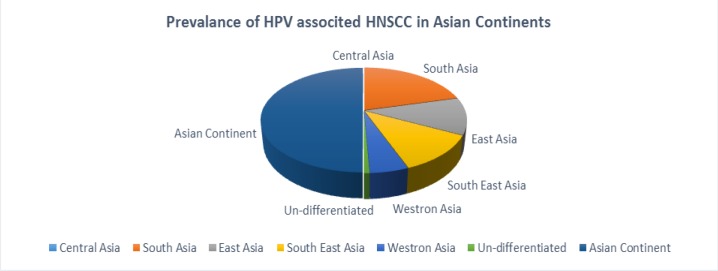
Comparative Analysis of HPV Distribution in Asian Continent

Reflecting 24 principal studies, overall (9 %) prevalence of HPV was recorded in South Asia, 5.04 % in South East Asia,4.93 % East Asia, 2.21 % in west Asia, 0.32% from a bilateral Pak-Chinese study and no relevant updated patients’ data was found from central Asian continent regarding HNSCC. From comparative point of regional HPV prevalence, highest prevalence of HPV was noted in south Asia, south East Asia and very less in central and west parts of Asian continent respectively (Figure 1).

*HPV-genotype Specific Prevalence*

Overall highest prevalence (10.08%) of HPV sub-genotype-16 was recorded in Asia among 3, 710 enrolled cases including 2,201 males, 1,149 females and 360 cases of unknown gender. While multiple HPV genotype prevalence was 5.5 % (204 cases), HPV-18 (2.12%), HPV-16 and 18 co-infection (1.51%) and 1.3 % cases were not known for any type specific HPV-genotype following whole Asian continent. Lowest percentage of HPV sub-genotypes 68, 72, 57, 39 were recorded respectively (Table 5). 

*Assessment of risk bias and clinical heterogeneity*

By following dichotomous data analysis random effect model at 95% CI. A significant clinical heterogeneity (Tau^2^ = 1.14, P< 0.00001, I^2^ = 87 %) was found between HPV positive HNSCCs and type specific HPV-16 led HNSCCs in Asian continent with an overall Odd ratio = 3. 41. (Figure 2A). Whereas, type specific HPV-18 clinical heterogeneity (Tau^2^ = 1.87, P< 0.00001, I^2^ = 81 %) with an Odd ratio of = 14.57 (Figure 2B). clinical heterogeneity following HPV co-genotype 16-18 in HNSCCs and HPV multi-genotype led HNSCCs (Tau^2^ = 5.43, P< 0.00001, I^2^ = 85 %) with an Odd ratio = 0.47 (Figure 2C). Comparing HPV-16 positive and HPV-18 positive genotype prevalence a significant heterogeneity was calculated (Tau^2^ = 3.52, P< 0.00001, I^2^ = 88 %). Greater heterogeneity in methodological aspect of included studies was also observed. Due to variable genotype testing methods including, PCR primers, ELIZA kits, ISH protocols and diversity in detection of HPV sub-genotypes followed by each individual study (Table 3). In parallel to determine the level of heterogeneity in our meta-analysis, we followed random effect model to estimate clear HPV type specific genotype prevalence in Asian continent (Table 5A- Table 5D).

The highest heterogeneity (Tau^2^ =10.70, P< 0.00001, I^2^ = 89 %, z = 5.09) was observed in overall 798 HPV positive cases in comparison to 48 non-determined HPV genotype case groups (Figure 2E). 

*HPV Sub-genotypes regional prevalence trends*

*South Asia*

In south Asia HPV specific genotype HPV-16, HPV-18, HPV-16,18 in combination and multiple HPV genotype were most prevalent 4% ,1.11% and 1.24% and 1.13% respectively. Pakistan ranked top reporting highest prevalence (2.72%) of HPV-16 and India in HPV-31(0.61%), (Table 2). 

*South East Asia*

HPV was (5.04 %) prevalent in south East Asia. However, 3.72 % multiple HPV genotypes were only reported from Singapore and overall HPV-16 was (1.24%) prevalent in South East Asia (Table 2). 

*East Asia*

In total 1,033 (27.84%) cases reported from east Asia HPV was found in 183 (4.93%) cases including (3.10%) HPV-16, 0.37% HPV-18, 0.03% HPV-33, HPV-39, HPV-58, HPV-66 and HPV-72 respectively. While 0.62 % cases were affected with multiple HPV genotypes. Taiwan was on top reporting (0.6%) prevalence of HPV-18 and in Japan HPV-35 was (0.08%) prevalent (Table 2).

*West Asia*

In western Asia 2.21% prevalence of HPV was estimated. Similarly, HPV-16 and HPV-18 genotypes were found most prevalent. Equal prevalence of HPV-16 was obtained from both Egypt and Turkey (Table 2). 

*Un-Categorized group*

A collaborative study conducted by Mujtaba et al., (2018) enrolled 506 HNSCC patients both from china and Pakistan. HPV was prevalent in 12 cases (0.32%). Inclusion of Mujtaba et al., (2018) in a distinct category was done to highlight recent progress in collaborative research culture as well (Table 2).

*Central Asia*

Unfortunately, as per criteria set forth, not a single study from central Asian territories were found including Turkmenistan, Kazakhstan, Kyrgyzstan, Uzbekistan and Tajikistan. 

**Table 1:** Detailed Inclusion and Exclusion Criteria

Inclusion Criteria	Exclusion criteria
Original Studies focusing HPV as ethology in HNC	Studies followed other than HPV role in HNC like, HPV role in cervical cancer
Studies reported from Central Asia, Middle East, East Asia, South Asia and western Asia.	Studies reported from other then Asian geographic regions
Clinical studies	Review articles, case reports, letter to the editor and short communication
Articles written in English language	Studies published in Other than English language
Articles who focused human subjects	Other than human subjects
Patients irrespective of age and gender	Studies reporting no relevant age and gender associated information.
Studies reported till 30^th^ August 2019.	Studies published before 1^st^ Jan 2007.

**Table 2:** Comprehensive Studies Selection Protocol Following Five Different Databases by Prisma

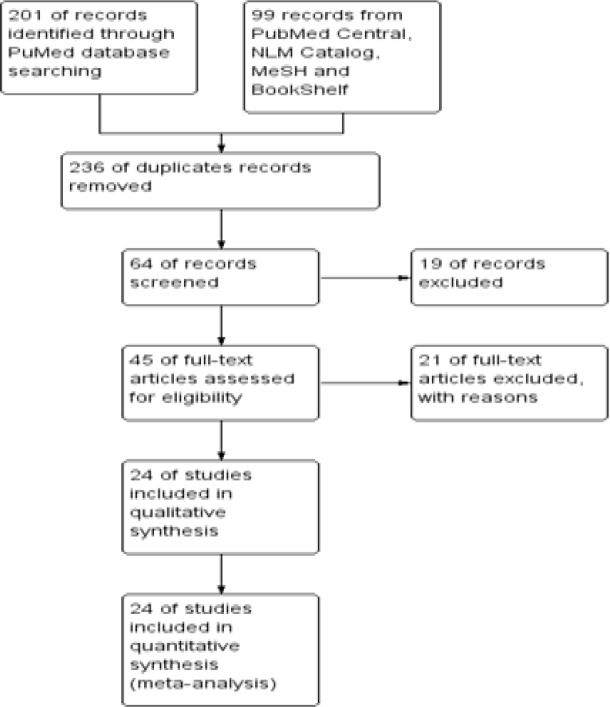

**Figure 2A:**
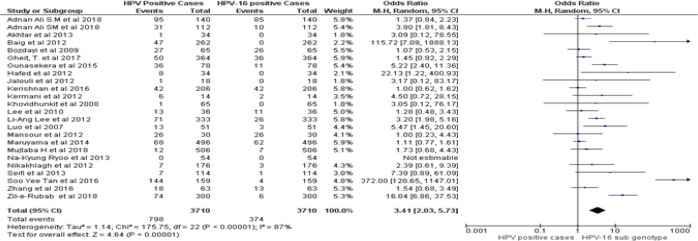
Forest Plot of Comparison, HPV Positive in HNSCCs and Type Specific HPV-16 Led HNSCCs

**Figure 2B:**
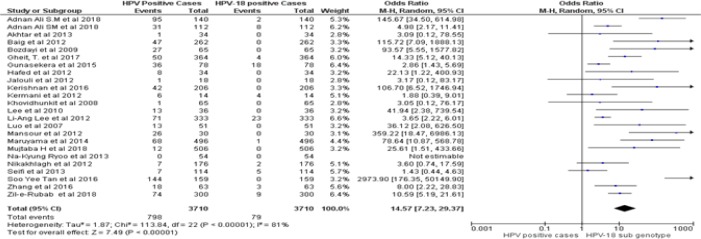
Forest Plot of Comparison, HPV Positive in HNSCCs and Type Specific HPV-18 Led HNSCC

**Figure 2C:**
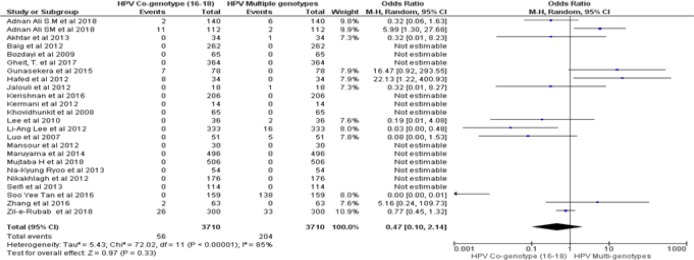
Forest Plot of Comparison, HPV Positive in HNSCCs and Multi -HPV Genotype Led HNSCCs

**Figure 2D:**
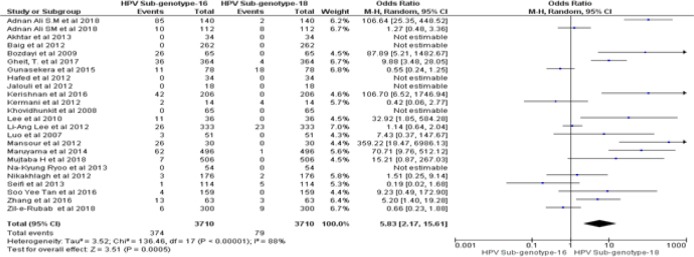
Forest Plot of Comparison, HPV-16 Positive in HNSCCs and HPV-18 Genotype Led HNSCCs

**Figure 2E:**
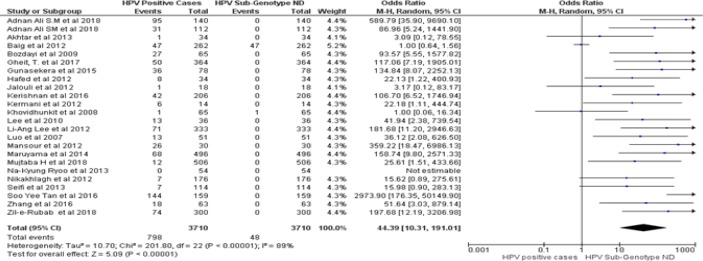
Forest Plot of Comparison, Positive in HNSCCs Cases and HPV-ND Genotype Led HNSCCs

**Table 3:** Characteristics of in Studies Included in Meta-analysis

Country	Study conducted	Year	No of positive cases	Total	Male	Female	Unknown	Mean Age Y	Method	Outcomes
Pakistan	Baig et al	2012	47	262	42	220	0	27	PCR	ND
Pakistan	Adnan Ali SM et al	2018	95	140	82	58	0	40	PCR	16,18
Pakistan	Adnan Ali SM et al	2018	31	112	76	36	0	47.8	PCR	16, 18, both
Pakistan	Zil-e-Rubab et al	2018	74	300	210	90	0	36	PCR	16, 18
India	Gheit, T. et al	2017	50	364	263	101	0	53.6	PCR	16, 18, 31, 35, 56
Singapore	Soo Yee Tan et al	2016	144	159	0	0	159	60.7	PCR	16, 18, 31, 45, 56, 68
China, Pakistan	Mujtaba H et al	2018	12	506	305	201	0	19	PCR	6, 11, 16, 58
Thailand	Khovidhunkit et al	2008	1	65	15	50	0	0	0	0
Taiwan	Luo et al	2007	13	51	48	3	0	55	PCR	16, 18, 33, 52
Taiwan	Li-Ang Lee et al	2012	71	333	316	17	0	51	PCR	16,18,52
Iran	Seifi et al	2013	7	114	52	53	9	31.6	PCR	6, 18, 66
Iran	Nikakhlagh et al	2012	7	176	151	25	0	67	PCR	16, 18,33,57
Iran	Kermani et al	2012	6	14	6	8	0	39.7	PCR	16,18
South Korea	Na-Kyung Ryoo et al	2013	0	54	0	0	54	2	ISH	ND
South Korea	Lee et al	2010	13	36	0	0	36	0	PCR	ND
Egypt	Hafed et al	2012	8	34	12	22	0	56	IHC	16,18
Egypt	Mansour et al	2012	26	30	15	15	0	52.9	PCR	16
Bangladesh	Akhtar et al	2013	1	34	0	0	34	0	PCR, IHC, ISH	ND
China	Zhang et al	2016	18	63	25	38	0	56	PCR	16, 18
Japan	Maruyama et al	2014	68	496	380	66	50	65	PCR	16, 18, 33, 35, 58
Malaysia	Kerishnan et al	2016	42	206	68	138	0	48.9	PCR	16
Sri Lanka	Gunasekera et al	2015	36	78	73	5	0	0	ELIZA	16,18
Turkey	Bozdayi et al	2009	27	65	62	3	0	58	ND	16
Yemen	Jalouli et al	2012	1	18	0	0	18	61.5	PCR	ND

ND, not determined.

**Table 4:** Comprehensive and Specific Data Retrieval Outcomes

No	Search Terms	Different Database Search Results					Overall Results
		PubMed	MeSH	PubMed Central	NLM Catalog	Book Shelf	
1	HNSCC in Asia	704	0	4,062	6	84	4,856
2	HPV in HNSCC	2,753	0	8,614	3	147	11,517
3	HPV subtypes in HNSCC	101	0	2,037	0	30	2,168
4	HNSCC aetiology	66	0	1,640	0	41	1,747
5	HPV vaccination	7,000	0	11,910	30	773	19,713
6	HPV in Central Asia	54	0	1,573	0	83	1,710
7	HPV in East Asia	58	0	1,124	0	47	1,229
8	HPV in South Asia	89	0	10,908	2	0	10,999
9	HPV in Weston Asia	0	0	17	0	0	17
10	HPV in Middle East	422	0	1,850	0	74	2,346

**Table 5:** Prevalence of HNSCC Linked HPV Sub-genotypes among Asian Population at 95% CI by Revman5.30

Country	"Positive cases"	Total	Male	Female	Other	"HPV 6"	"HPV 11"	HPV 16	"HPV 18"	"HPV 31"	"HPV 33"	"HPV 35"	"HPV 39"	"HPV 45"	"HPV 52"	"HPV 56"	"HPV 57"	"HPV 58"	"HPV 66"	"HPV 68"	"HPV 72"	"Both 16,18"	"Multiple genotype"	ND
South Asia																								
Pakistan	247	814	410	404	0	0	0	101	19	0	0	0	0	0	0	0	0	0	0	0	0	39	41	47
India	50	364	263	101	0	0	0	36	4	6	0	2	0	0	0	2	0	0	0	0	0	0	0	0
Bangladesh	1	34	0	0	34	0	0	0	0	0	0	0	0	0	0	0	0	0	0	0	0	0	1	0
Sri Lanka	36	78	73	5	0	0	0	11	18	0	0	0	0	0	0	0	0	0	0	0	0	7	0	0
Total	344	1290	746	510	34	0	0	148	41	6	0	2	0	0	0	2	0	0	0	0	0	46	42	47
"Relative %HPV Prevalnce in south Asia"	9	34/77	20/11	13/75	0/92	0	0	4	1/11	0/16	0	0/05	0	0	0	0/05	0	0	0	0	0	1/24	1/13	1/27
South East Asia																								
Singapur	144	159	0	0	159	0	0	4	0	0	0	0	0	2	0	0	0	0	0	0	0	0	138	0
Thailand	1	65	15	50	0	0	0	0	0	0	0	0	0	0	0	0	0	0	0	0	0	0	0	1
Malaysia	42	206	68	138	0	0	0	42	0	0	0	0	0	0	0	0	0	0	0	0	0	0	0	0
Total	187	430	83	188	159	0	0	46	0	0	0	0	0	2	0	0	0	0	0	0	0	0	138	1
"Relative %HPV Prevalnce in south East Asia"	5/04	11/59	2/24	5/07	4/29	0	0	1/24	0	0	0	0	0	0/05	0	0	0	0	0	0	0	0	3/72	0/03
East Asia																								
China	18	63	25	38	0	0	0	13	3	0	0	0	0	0	0	0	0	0	0	0	0	2	0	0
Japan	68	496	380	66	50	0	0	62	1	0	1	3	0	0	0	0	0	1	0	0	0	0	0	0
South Korea	13	90	0	0	90	0	0	11	0	0	0	0	0	0	0	0	0	0	0	0	0	0	2	0
Taiwan	84	384	364	20	0	0	0	29	23	0	0	0	1	0	8	0	0	0	1	0	1	0	21	0
Total	183	1033	769	124	140	0	0	115	27	0	1	3	1	0	8	0	0	1	1	0	1	2	23	0
"Relative %HPV Prevalnce in East Asia"	4/93	27/84	20/73	3/34	3/77	0	0	3/1	0/73	0	0/03	0/08	0/03	0	0/22	0	0	0/03	0/03	0	0/03	0/05	0/62	0
West Asia																								
Iran	20	304	209	86	9	0	0	6	11	0	1	0	0	0	0	0	1	0	1	0	0	0	0	0
Egypt	34	64	27	37	0	0	0	26	0	0	0	0	0	0	0	0	0	0	0	0	0	8	0	0
Turkey	27	65	62	3	0	1	0	26	0	0	0	0	0	0	0	0	0	0	0	0	0	0	0	0
Yemen	1	18	0	0	18	0	0	0	0	0	0	0	0	0	0	0	0	0	0	0	0	0	1	0
Total	82	451	298	126	27	1	0	58	11	0	1	0	0	0	0	0	1	0	1	0	0	8	1	0
"Relative %HPV Prevalnce inWest Asia"	2/21	12/16	8/03	3/4	0/73	0/03	0	1/56	0/3	0	0/03	0	0	0	0	0	0/03	0	0/03	0	0	0/22	0/03	0
Un-Categorlzed																								
China, Pakistan	12	506	305	201	0	2	2	7	0	0	0	0	0	0	0	0	0	1	0	0	0	0	0	0
Relative % Prevalnce in Pak-China	0/32	13/64	8/22	5/42	0	0/05	0/05	0/19	0	0	0	0	0	0	0	0	0	0/03	0	0	0	0	0	0
Total ASIA	798	3710	2201	1149	360	3	2	374	79	6	2	5	1	2	8	2	1	2	2	0	1	56	204	48
"Relative %HPV Prevalnce Asia"	21/51	100	59/33	30/97	9/7	0/08	0/05	10/08	2/13	0/16	0/05	0/13	0/03	0/05	0/22	0/05	0/03	0/05	0/05	0	0/03	1/51	5/5	1/29

## Discussion

First time our review critically analyzed the available literature covering the whole Asian continent and reported 21.5% prevalence of HPV associated HNSCCs with clear heterogeneity insights. While prior to current study HPV association in HNSCCs was considered in the range of 12.8%-59.9 %, including HPV-35, HPV-33, HPV-31, HPV-18 and HPV-16 genotypes only. (zur et al., 2009; Westra et al., 2009; Liebertz et al., 2010; Nasman et al., 2009). However, our review was a collaborative research effort to minimize cancer research gap in Asian continent and focussed prevalence of more than fifteen HPV sub-genotypes. Similar large scale regional and worldwide studies covering whole Asia reported (33%) HPV prevalence in HNC-subsite (Oral cavity) among Asians, 16% in European and 16. 1% North American populations respectively (Kreimer et al., 2005) In 2014 another review reported by Abogunrin S et al focusing European population estimated 40 % prevalence of HPV in head and neck carcinomas. We accept exact prevalence of HPV associated HNSCCs among Asian population is still debateable and our study has certain limitations including non-availability of homogenise reported studies from Asian sub-continents especially central Asia, western Asia and even from south and south east Asia demand large scale collaborative research culture to standardize HPV led HNSCCs aetiology. Beside very potential outcomes, our review has certain limitations including a significant level of heterogeneity in comprised studies, Publication year, variable number of patients, specimen type and HPV detection methods. Similarly, the possibility of confounder cannot be ignoring, because some studies evaluated more then one type of HPV sub-genotype. while an included study detected HPV-16 genotype only as well. 

In conclusion, highest priority should be given to initiate homogeneity cancer research programs among Asian countries especially; Afghanistan, Iraq, Nepal and Yemen to assess the tumour positivity rates of HPV in HNSCCs. While in countries like Pakistan, India, Bangladesh, Thailand, North Korea, Iran, Japan and Turkey more collaborative research is needed to standardize prevalence of HPV associated HNSCCs across Asian Continent.

## Conflict of Interest

Nil. 

## Abbreviations

PCR, Polymerase chain reaction; ISH, in-situ-hybridizations; ELIZA, Enzyme linked immunosorbent assay; HPV, human papilloma virus; HNC, head and neck cancer; CNS, Central Nervous System. 
